# The Gordon Memorial Lecture: genotype, phenotype, selection and more: improving the skeletal health of laying hens

**DOI:** 10.1080/00071668.2025.2460054

**Published:** 2025-03-07

**Authors:** I. C. Dunn

**Affiliations:** The Roslin Institute and Royal (Dick) School of Veterinary Studies, University of Edinburgh, Edinburgh, UK

**Keywords:** Genetic selection, reproduction, bone, long life layer, welfare

## Abstract

1. This review is a comprehensive exploration of the author’s work in improving skeletal health in laying hens, focusing on the insights from genetics on nutritional, and environmental factors. It discusses the importance of the large number of disciplines that have contributed to the efforts to tackle bone quality in laying hens, particularly the keel bone.

2. The transition from cages to non-cage environments has increased keel bone damage, despite improving overall skeletal health. It is a welfare paradox that improving the hen’s environment has often been accompanied by greater skeletal damage.

3. The role of genetics has been important in understanding and addressing bone health issues and will be a major factor in their improvement. This includes the identification of specific genes, like cystathionine-β-synthase, which has led to nutritional interventions using betaine supplementation to improve bone quality by targeting the one carbon pathway.

4. The role of the timing of puberty and its genetic control is an additional factor in bone health, and new methods of measuring bone density in live birds are now important to monitor potential issues and deliver genetic solutions.

5. The review emphasises a multi-faceted approach, combining genetics, nutrition, rearing practices, and housing design is required in order to improve skeletal health and enhance the welfare and sustainable performance in laying hens.

## Introduction

As a poultry scientist, it was a great honour to give the 2024 Gordon Memorial Lecture. This review looks at the issue of bone quality in laying hens, predominantly from genetic viewpoint. That is not to say that it only considers genetics, it additionally discusses solutions that have been inspired by the insights genetics have provided.

The use of genetics has proved to be extremely illuminating, both for understanding the physiology of the trait and in providing solutions, both directly and indirectly. This review tackles some of the dogma that surrounds the issue of laying hen bone quality which has, in the past and may in the future, get in the way of finding solutions. It focusses on the facts, rather than the beliefs. Hence, this review discusses the issue of bone quality in laying hens from a predominantly genetic viewpoint.

## Why I am a poultry scientist and how did I get here?

I want to start first with why I am a poultry scientist and how I got to where I am now. I started straight from school as an assistant scientific officer working at the Poultry Research Centre in Edinburgh (University of Edinburgh Poultry Research Centre 1947-1986), which was then an Institute of the Agricultural Research Council. I had the simple ambition to work in biology and, although perhaps not an orthodox approach, it has fortunately worked for me. I worked initially on laying hen urolithiasis (Blaxland et al. 1980) and deep pectoral myopathy in breeding turkeys and broilers (Martindale et al. 1979). At that time, whilst learning scientific methods regarding physiology, clinical chemistry, biochemistry and anatomy and seeing it applied, I became convinced I wanted to become a scientist. It was particularly striking to understand the aetiology of deep pectoral myopathy, going from the belief this was an infectious disease to demonstrating that it had an anatomic and metabolic basis. Working at that time with people that were really inspiring with a broad interest in science and beyond was a great stimulus.

After completing a degree in Physiology and Pharmacology at St Andrews University at the expense of the Agricultural and Food Research Council it was a condition of their largesse that I had to return to work in that organisation. I wanted to study for a PhD, but funding was being heavily cut at that time and so I instead worked on aspects of avian endocrinology and photoperiodism (Dunn and Sharp 1990, 1992; Dunn et al. 1990; Goddard et al. 1988; Sharp et al. 1987). Eventually, with persistence, I completed a PhD in molecular biology. This was a fairly new area at that time and, if it had not been for the advent of thermostable Taq and the polymerase-chain reaction (PCR; Saiki et al. 1988) that is almost a household name after Covid 19, I might still be trying to complete that PhD. The objective was the characterisation of avian gonadotropin releasing hormone gene, which is at the centre of the brain’s control of reproduction and which was unknown at the time (Dunn et al. 1993). The direction of travel in this field was to ultimately produce transgenic poultry, and I spent a year at Harvard working with GnRH transgenic reporter mice and cell lines to understand this approach (M. Zakaria et al. 1996; M. M. Zakaria et al. 1996). However, after Dolly the sheep’s entry on to the scene at the now named Roslin Institute (Wilmut et al. 1997), the Ministry of Agriculture Fisheries and Food, who were funding my research, started to distance themselves from transgenic animals. I then metamorphosed into more of a population geneticist rather than a molecular geneticist. So, serendipity has played a large part in the situation I find myself now, but, on this journey, I have gathered a range of scientific insights and methods that have enabled me to generate discoveries from genetics and utilise the results to deliver understanding and solutions. I may be a master of none of these disciplines, but I hopefully know something about each of them! Serendipity played a further role in my work on bone health. Colin Whitehead had, for some time, been working on bone health with Dietmar Flock at the Lohmann breeding company and with Heather McCormack, Bob Fleming and Steve Bishop (Bishop et al. 2000; Fleming et al. 1994, 1996, 1997). I had been working with Lohmann on egg quality genetics (Bain et al. 2009; Dunn et al. 2005, 2009) so, when the opportunity came to use population genetics to discover genetic loci affecting bone quality, it was very timely (Dunn et al. 2007).

## Why has laying hen bone health become such an important topic?

It is important to first consider why bone health in laying hens has become such an important issue. In fact, it has always been an issue and, from publications going back many years, it is not new (Darwin 1868; Gregory and Wilkins 1989; Harms and Arafa 1986; Urist and Deutsch 1960; Warren 1937). Arguably, it is obvious that laying hen bone health was always going to become more of an issue as hens were moved out of traditional cages into a more diverse, but challenging, environment (Fleming, McCormack, et al. 2004). Of course, the move into cages, which occurred around 80 years ago, and the recent move away from cages both were done for logical reasons. *i.e.*, the control of the environment and disease and then the fulfilment of natural behaviours. But both transitions have had their consequences.

Recently the problem of keel bone health has reached the European Parliament (European-Parliament 2021), resulting in questioning of breeding practices for laying hens. There is no doubt that a significant number of laying hens suffer from damage to their skeleton and, for the most part, this involves the keel or sternum, especially in complex alternative systems (Sandilands 2011). Hens in multi-tier housing have more damage to their keel bones than those in single tier housing for example (Marggraff et al. 2024). The issue is also seen in free-range organic production and, although the fracture estimate was low in one study, deviations are frequent (Göransson et al. 2023). However, an earlier study listed a range of damage and fractures in organic farming across Europe and identified the cause to be multifactorial, with daylight as the most important factor (L. Jung et al. 2019).

**Figure 1.**
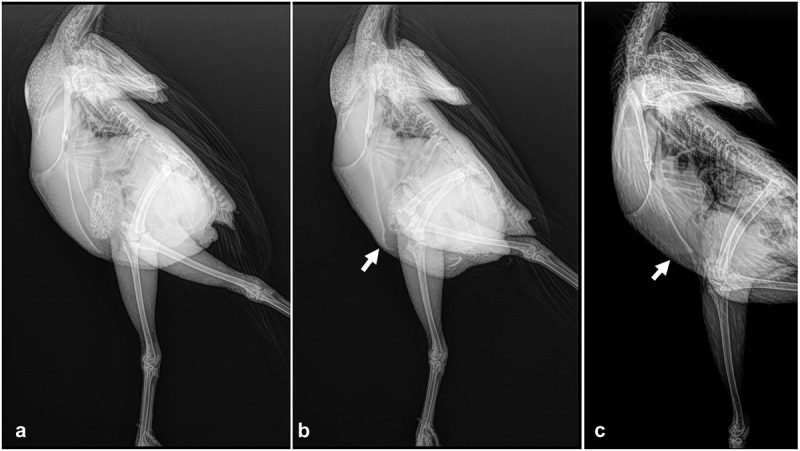
Three x-rays of living hens showing (a) a hen during the laying period with a normal keel, (b) a hen during the laying period with a distorted keel bone at the caudal tip of the keel which is indicated with an arrow, and (c) a pre-pubertal hen at 14 weeks of age showing the limited extent of the keel bone mineralisation with an arrow showing the caudal most extent of mineralisation.

A radiograph of the laying hen (Figure 1, panel B compared to panel A) demonstrates the damage that can occur. However, some care needs to be taken in studies on prevalence, as it is important to distinguish *post-mortem* damage from injuries occurring in the farm. Depletion damage is likely to be an important contributor to the total. This needs to be distinguished, as different ameliorative approaches are needed for various periods in the life of a hen where damage can occur (Gregory and Wilkins 1989; Gregory et al. 1990; Knowles et al. 1993).

The keel is the major attachment point for muscles which are needed to provide for flight. In particular, the pectoralis muscles provide lift, whilst the supracoracoideus muscle is responsible for the upstroke (Biewener 2011). Because of the limited role of flight in domestic chickens, Darwin’s hypothesis may be appropriate, which states that those structures that become redundant or less used will diminish physically in a structural or functional sense. Darwin used the keel as an example of a structure that was diminished due to the lack of requirement for flight in domestic chickens. Of course, chickens do fly to some extent (Leblanc et al. 2018) and so it is difficult to say whether the hypothesis is completely correct. However, flight is not commonly practiced by domestic hens although many commercial breeds are known to fly short distances (Colson et al. 2008), although some flights end in collisions (Campbell et al. 2016). Personal observation confirms that some hens seek out the highest points they can reach in their environment. Whether this is more or less than the wild living jungle fowl’s ascent to roosting sites is debateable (Collias and Collias 1967). Certainly, the sternum is late to mineralise (Buckner et al. 1947; Figure 1 panel C) and is proportionally diminished in size in comparison with the native jungle fowl. Overall, the lack of natural selection on the keel may be in part why there is so much damage to that structure, whatever the hens background, highly selected or not (Jung et al. 2024).

Research has tried to answer both questions about what does and does not cause the problems of skeletal health. Because for the foreseeable future, the demand for protein in the world is not going to diminish and eggs are a near perfect source of protein for human nutrition (Rehault-Godbert et al. 2019). Thankfully more research is coming on stream that is looking for solutions rather than solely reporting prevalence. The remainder of this paper will focus on the areas where we have contributed to solutions to ameliorate skeletal damage and, in particular, to the keel.

## The welfare paradox

### The benefits and negatives of non-cage environments

One reason why the problem of keel bone damage has become much more prevalent seems to be the transition from cages to non-cage environments. In many countries, this has been due to legislation, as is the case in the EU or the U.S.A., through retailer or consumer pressure. Although many people do not believe that change will proceed at the same speed in the future, the evidence suggests that, in many countries, the perception of welfare and how cages are not desirable have gained traction with consumers (Gautron et al. 2021).

Paradoxically, keel bone quality and, indeed, general skeletal health is actually improved by being in extensive non-cage environments, certainly towards the end of production (Fleming et al. 2006; Fleming, McCormack, et al. 2004). This has been shown repeatedly because the loading of the skeleton from wing flapping or walking increases the density and strength of the bones (Jendral et al. 2008; Johnsson et al. 2023; Regmi et al. 2015; Rodriguez-Navarro et al. 2018). Unfortunately, some, but not all, extensive systems result in greater complexity in the environment and, in aviary situations which are multi-tiered, the environment can be actually complex (Rufener and Makagon 2020; Sandilands et al. 2009). Aviaries can have many levels and tiers with metal structures, perches that birds have to negotiate, as hens certainly like to use the different elevations within aviaries (Stratmann et al. 2015). There appears to be increased chance of skeletal damage as hens transition from level to level (Stratmann et al. 2015). Certainly, the number of high acceleration events a hen has correlates with keel bone damage (Baker et al. 2024).

What has occurred is a classic welfare paradox. The clear advantages to the welfare of the hen in non-cage environments are balanced with the increased risk of damage to the keel. To tackle this paradox, it is possible to take a number of approaches. The constructed environment can be adapted to reduce the damage of transitions (Stratmann et al. 2015). The bird itself could be bred to have a stronger skeleton and there are behavioural traits that could potentially be selected (Baker et al. 2024). Nutrition must also be considered, and, although a lot is known about the nutritional factors that are important for strong bones, it must be ensured that those are being supplied to the bird (Bouvarel et al. 2011). There may be aspects of skeletal health that have not been considered sufficiently in the nutrition of hens that could still be optimised. There is no doubt that there are other factors in the management of laying hens in terms of their adaptation to the rearing environment and how it interacts with their move to the laying environment (Casey-Trott et al. 2017; Nazar et al. 2022). There are many ways that can be employed to ameliorate such issues. However, it is almost certain that it will never be eliminated completely, but it can be reduced.

## What is special about avian bone?

In many respects, the generation of avian bone (Pines and Reshef 2015) is similar to bone growth in mammals (Salhotra et al. 2020). The balance between osteoblasts and osteoclasts maintains bone structure during growth. In the appendicular skeleton or long bones, concentric growth of bone takes place with the successive modelling and remodelling of the bone by osteoblasts and osteoclasts (Salhotra et al. 2020). At the tips of the bone where growth occurs to extend the bone, osteocytes differentiate into osteoblastic cells at the growth plate (Pines and Reshef 2015). The osteoblasts lay down the organic matrix, which is predominantly collagen, which is cross-linked to give it a strong structure. On this organic matrix scaffold, minerals, predominately calcium phosphate, are deposited by the action of the osteoblasts (Pines and Reshef 2015).

### Medullary bone

Where avian bone does differ markedly from the bones of other clades is in the formation of medullary bone (Vandevelde et al. 1984). This is specialised bone that is adapted for laying eggs and ensures that the demands for calcium to produce the egg shell can be met whilst maintaining calcium homoeostasis (Dacke et al. 1993). It forms in a number of places in the skeleton, specifically the long bones, and is found in the sternum, although in smaller quantities (Benavides-Reyes et al. 2021). It is a woven structure, lacking the strength of cortical bone, but it does contribute to strength to some extent (Fleming et al. 1998). It has evolved to be rapidly deposited and reabsorbed. Indeed, this happens to some extent every 24 h when a hen lays an egg, with the structure being amenable to rapid mineral resorption (Kerschnitzki et al. 2014; Vandevelde et al. 1984). The medullary bone is less dense and has a relatively small crystal size which may promote rapid reabsorption, although other aspects of its structure are likely important (Dominguez-Gasca et al. 2019; Kerschnitzki et al. 2014). In fact, the biology of medullary bone seems particularly fascinating to me. Although there have been a number of studies on its biology, perhaps because it is a uniquely avian structure, these are not huge in number (Dacke et al. 1993). Certainly, modern genomic approaches have not been successfully applied to understand its function. What does not seem to be known is why the activity of osteoblasts and osteoclasts apparently shifts to the medullary bone and its production. It is possible to induce bone formation by giving birds oestrogen which mimics the situation when birds start to lay eggs (Miller and Bowman 1981). Beyond this, little is understood of what programmes the spatial positioning of the medullary bone structure and, at least for academic research and potentially for improving the quality over the life of the hen, it is something that needs more attention. What has been clear for a long time, is that adaptation to laying eggs and the shift to medullary bone production is why the skeleton of the hen is more prone to damage. Little or no damage is seen in males (Fleming, McCormack, et al. 2004) and, if you inhibit reproduction in females, they have stronger bones with little damage (Eusemann, Sharifi, et al. 2018).

## Is keel bone damage because hens lay too many eggs?

However, it needs to be pointed out that it has been clearly demonstrated that it is not the number of eggs that hens lay that is an issue (Dunn et al. 2021), it is simply the fact that they lay eggs at all. That is an important distinction. This is one of the dogmas that has surrounded the area of skeletal health in laying hens and has led to a belief that just reducing egg production is a panacea. The standpoint that it is the number of eggs a hen lays has just become shorthand for bone problems in laying hens and may have hampered work to find solutions. Looking at historical data on pedigree flocks of hens, there were no genetic correlations between a range of bone quality traits and the number of eggs hens laid (Dunn et al. 2021). When you look in the literature, of course there are publications that compare lines of hen that lay more eggs than ones that do not where the latter have better bones (Eusemann, Baulain, et al. 2018; Habig et al. 2021). But equally there are examples, perhaps not surprisingly given the history of keel bone damage, that have similar or greater bone damage even with lower egg production (L. S. Jung et al. 2024). It is to be hoped that the focus of research moves on from an assertion that it is the number of eggs laid and moves on to where there is sound proof for relationships with other traits, which may be causal.

## Genetics suggest puberty

Genetic studies have demonstrated that it is not the number of eggs laid, rather the timing of puberty, measurable as the onset of egg laying, is clearly important for determining skeletal health, at least in one of the lines that was studied (Dunn et al. 2021). In other words, it was not the number of eggs produced but when the birds came in to lay. But at the same time in another line, which laid brown eggs, any effect of puberty was not apparent (Dunn et al. 2021). Therefore, care is needed for stating puberty is a factor in all situations. Surprisingly, there are only a limited number of examples of where puberty has been recognised as a factor for bone quality in laying hens (Gebhardt-Henrich and Frohlich 2015). There are some trials where this has been tested and not found to be statistically important for a measurement of skeletal health, even if bone length was altered (Hester et al. 2011). This might be because, in modern laying hens, manipulation of puberty by photoperiod alone tends to have quite small effects, whilst treatments that are unlikely to be compatible with maintaining long-term egg production achieve, at best, 14 d delay (Lewis et al. 1998).

## A nutritional solution suggested by genetics

This section considers how genetics has contributed to a potential nutritional solution to improving bone health in laying hens. Decades of work have been devoted to understanding the genetic contribution to bone quality in laying hens (Bishop et al. 2000). Then to isolating specific regions of the chromosome that are responsible for larger parts of the variation of genetic origin using crosses of lines retrospectively selected for bone quality (Dunn et al. 2007). As the pace of genetic tool delivery has quickened, association studies have been possible using single nucleotide polymorphisms (SNP) that fine-map the loci in the pure line and allow tracking through successive generations (De Koning et al. 2020). One genetic region has been pinpointed with a relatively small number of genetic markers that defined two different haplotypes. It has been possible to use bone samples from haplotypes that had a large difference in bone quality to compare the expression of genes, so-called transcriptomics. Having access to large populations has enabled the control of variables that are known to alter bone quality. This includes time of day, which influences egg formation and, therefore, the status of the bone, in order to get hens that were similar physiologically but their genotype at the genome location differed. Only a small number of genes were different between the two genotypes. One of them stood out in terms of its significance and that was an enzyme cystathionine beta synthase (De Koning et al. 2020). This gene was located within the region of the chromosome that had already identified as being where the effect was located. This was strong evidence that this gene was likely to be causative for the observed difference in bone quality between the genotypes and its substrate, homocysteine, was found to differ between the haplotypes (De Koning et al. 2020). Homocysteine is known in hindsight to potentially have effects on bone but cystathionine-β-synthase was not a gene that would necessarily have ever been identified as being a cause of differences in bone quality.

Cystathionine-β-synthase is an important component of the one carbon pathway, as it is central to cell metabolism (Jhee and Kruger 2005). Its substrate, homocysteine, is an amino acid, but is not used directly in protein synthesis. It does, however, inhibit the actions of cross linking between cysteine bonds by inhibiting lysyl oxidase expression and enzyme activity through formation of homocysteine thiolactone (Liu et al. 1997). Cross linking is important for the quality of collagen (Knott et al. 1995). The collagen matrix is the structure in which mineralisation occurs and gives plastic or ductile properties to bone (Knott and Bailey 1998).

What can be done with this information? Most studies go no further than listing what genes may differ between different states. Few studies have identified a causative gene for a trait. In this case, it has been possible to both demonstrate a functional effect and provide a potential nutritional solution. Cystathionine-β-synthase is involved in the conversion of homocysteine into cystathionine as part of the one carbon pathway and, given the large amount of biochemistry in this pathway, it is possible to manipulate it to reduce its concentration (Stipanuk 2004). The re-methylation of homocysteine into methionine, which is a limiting amino acid, should reduce homocysteine concentrations. Betaine, or trimethylamine, is a proven feed additive that facilitates the re-methylation of homocysteine to methionine which reduces the concentrations of homocysteine. The hypothesis that reducing homocysteine by feeding betaine would improve bone quality has been tested. The concentration of homocysteine was reduced and bone breaking strength and density was higher when hens were fed betaine (Maidin et al. 2021). Going from basic genetics, it has been possible to work through to a relatively inexpensive method to improve bone quality.

## What about direct genetic improvement?

Two indirect ways that genetics can help bone health in laying hens include nutrition and age at puberty. What has not been discussed is how direct selection for bone health could be achieved. This might seem quite straightforward, especially as most studies have shown that genetics accounts for 40% of the variance in the trait (Bishop et al. 2000; Dunn et al. 2007, 2021). But the reality is that there has not been a practical method to measure bone quality that could be applied at a scale that is needed in commercial breeding flocks. Most importantly, the method must keep the hen alive to subsequently breed from. The evidence that there is a genetic component to bone quality in laying hens has been derived from using destructive methods. In other words, the birds have had to be killed to dissect the bones to then make measurements of density or breaking strength.

It is not for the want of trying to find such methods. Over the years, the Roslin Institute has investigated a number of possible ways in which this could be done. Ultrasound using the toes and a marker was tried and, indeed, worked (Fleming, Korver, et al. 2004) but appeared not to have a high correlation with *post-mortem* traits. Ultrasound was subsequently tried on the humerus, which was successful and heritable, but there was no genetic correlation with any bone trait whatsoever, other than medullary bone score and, hence, this work has remained unpublished. Others have used CT scans (Donko et al. 2018) or DEXA (Schreiweis et al. 2005) which are all x-ray-based methods but they tend to be expensive and require time for image acquisition. Using new advances in technology, specifically the digital capture of two-dimensional radiographic images, has proven viable (Wilson et al. 2023). This is rapid, taking seconds to capture an image and is less than a minute, including weighing and taking a hen to and from a holding crate. A method has been devised to make a density estimate of the tibiatarsus from the image that can be used for genetic evaluation. This live bird density estimate correlates with bone quality as well as existing *post-mortem* measurements (Wilson et al. 2023). Most importantly, this estimate has good genetic heritability and correlation with *post-mortem* measures of bone quality and across the lifetime of the hen (Andersson et al. 2023).

There are other ways that have been tried with live hens and palpation of the keel bone is probably the most obvious, as it is cheap and can target the bone that is most in question for damage. Palpation detects deviations and obvious fractures in the keel and it is relatively easy to do (Rufener et al. 2018). However, data from palpation require a large degree of chance events to occur, such as the hen having a collision to manifest a phenotype. This may vary enormously with the environment and aspects of the phenotype are a binary product of chance events, rather than a continuous variable. There are also problems of inter-observer variation because of its subjectivity (Tracy et al. 2019). Nonetheless, it does allow the detection in the field with no equipment required (Marggraff et al. 2024).

## What about the keel bone?

If a quantitative measure of skeletal health is better then why not a direct measure of the keel bone? The methods previously outlined measure the tibiatarsus for a number of reasons. It is easier to get a density estimate of the tibiatarsus in a live bird with some compensation for the background effect of surrounding tissue (Wilson et al. 2023). Bone density has been repeatedly shown to be related to aspects of skeletal health, including bone strength (Dunn et al. 2021; Fleming et al. 1994; Schreiweis et al. 2003). In the case of the keel, there is a large quantity of overlying pectoral muscle. This is not easy to subtract successfully on a live bird X-ray to give a good density estimate and the keel is quite thin, so the signal-to-noise ratio is high. Instead, efforts have been made to demonstrate that keel bone quality correlates with the quality of the rest of the skeleton and, in particular, the tibiatarsus properties (Maidin et al. 2024). This is important because the keel is viewed as somehow different from all other bones. It does have some differences from the rest of the skeleton, such as it mineralises later (see Figure 1 panel C) and its mineral density declines from the cranial to caudal tip (Benavides-Reyes et al. 2021). As Darwin hypothesised, the keel may well be a structure which is no longer so important for domestic chickens and may have diminished in size due to an absence of selection pressure (Darwin 1868). Any evidence that the keel has more damage when birds have poorer overall skeletal quality has been obscured by the paradox that, due to fracture callus formation, the worst quality keels have higher density on radiographs (Maidin et al. 2024). However, when *post-mortem* scoring of the keel is made, it is clear that the worse the quality of a bone such as the tibiatarsus, the worse the keel bone scores (Maidin et al. 2024). Such efforts have shown that measuring and basing genetic improvement on skeletal quality measurement, such as tibiatarsus density, will result in improvements in the keel. Skeletal density is not affected by chance events, such as collision, and should be more reliable than subjective scores of the keel itself.

## Conclusions

The review presents a genetic perspective on improving bone quality, and direct genetic selection can work. A practical phenotype is key to progress, and there is finally one method based on tibiatarsus density on live hens (Wilson et al. 2023). This may need further development or simplification. Efforts are being made to automate aspects of the process, and it is hoped that poultry breeders will embrace the opportunity to reduce bone damage with this or better methods. This will be an important step in improving the future sustainability of the industry.

Research to improve bone quality should target traits that have good evidence for being important and measurable. Bone quality can be measured directly, although correlated traits, such as age at puberty or body weight, which is, in part, a consequence of age at puberty, are all measurable and have known effects on bone quality. There is little proven support for the hypothesis that increased number of eggs produced by modern hens is the cause of poor bone quality.

Using genetics to find the genes controlling bone quality is not a waste of time if it brings knowledge of function which might lead to new solutions. Cystathionine-β-synthase has led to new nutritional interventions and there are many other genome locations awaiting exploration for controlling bone quality (Podisi et al. 2012; Raymond et al. 2018; Sallam et al. 2023). The Nobel Prize winning biologist Sidney Brenner said ‘there is a gene for every scientist’, and there is certainly a genetic loci for every scientist. Understanding the consequences of these loci will potentially bring resolutions to important issues. The genetic markers associated with cystathionine-β-synthase alone could improve flock bone strength by 6–18% (De Koning et al. 2020) and there is plenty evidence that genomic selection would be possible for bone quality which can help make the task easier as this technology is now widely used.

Genetics has provided solutions, but it is not the only solution, as rearing, nutrition and housing design are all important. A comprehensive approach will be needed to optimise the welfare of the future ‘long life layer’ under conditions the consumer and retailers are increasingly demanding.
